# Research on the influence mechanism of creative time pressure on employee knowledge hiding: Evidence from creative service enterprises in China

**DOI:** 10.3389/fpsyg.2022.937304

**Published:** 2022-07-27

**Authors:** Xiaoxia Chen, Wenhe Lin, Anxin Xu

**Affiliations:** ^1^Jinshan College of Fujian Agriculture and Forestry University, Fuzhou, China; ^2^College of Economics and Management, Fujian Agriculture and Forestry University, Fuzhou, China

**Keywords:** creative time pressure, work passion, knowledge hiding, team psychological safety climate, affective events theory

## Abstract

Employee knowledge sharing is critical to the success of creative service enterprises. However, knowledge hiding is prevalent in creative service enterprises. Using 381 advertising agency employees as respondents, we explored the mechanism of action of creative time pressure affecting knowledge hiding. We constructed a regulated dual-path model by drawing on affective event theory, with work passion as a mediating variable and team psychological safety climate as a moderating variable. The results show that creative time pressure increases employees’ knowledge hiding; creative time pressure mitigates knowledge hiding through the effect of harmonious passion, while obsessive passion enhances employees’ knowledge hiding; team psychological safety climate can regulate the relationship between creative time pressure and two types of work passion and the strength of the two paths. Therefore, the mediating effect of harmonious passion is stronger in a high team psychological safety climate, while the mediating effect of obsessive passion is stronger in a low team psychological safety climate.

## Introduction

Creative service enterprises refer to enterprises that meet the needs of customers through the creative services of employees and use them as the driving force for their own development (Howkins, 2013), such as advertising companies, cultural and art service companies, media companies, digital technology companies, etc. It has the characteristics of knowledge-intensive, creative, differentiated, and technical. Different from other types of enterprises, creative service enterprises take creativity and intellectual capital of employees as the main input, and engage in knowledge-based creative activities. The knowledge factor is particularly important in creative service enterprises. Employees, as the core carriers of knowledge and owners of creative capital, bear heavy responsibility for knowledge acquisition, transfer, and utilization. However, due to the complexity of knowledge, the cost of acquiring knowledge increases gradually, making employees engage in knowledge hiding to maintain their uniqueness and irreplaceability in the content department of the organization (Connelly et al., 2011). A total annual productivity lost due to knowledge hiding were reported to cost up to $ 47 million for large business in a study of >1000 employees (Panopto, 2018). Employees also wasted 5.3 h per week waiting for knowledge, which slows down organizational productivity (Panopto, 2018). Studies have shown that knowledge hiding hinders the exchange and flow of knowledge by constructing “information barriers,” negatively affecting creative team members’ cooperation and creativity (Tadić et al., 2014; Rhee and Choi, 2016), innovation behavior (Zhang and Wang, 2021), and team performance (Zhang and Min, 2019) at all levels of the organization, and this phenomenon is common in knowledge-based organizations (Singh, 2019; Chatterjee et al., 2021; Nguyen et al., 2022). The prevention of knowledge hiding, the realization of organizational knowledge sharing, and the promotion of the transformation of individual knowledge to the organizational level are important tasks in current knowledge management.

The knowledge hiding of employees in creative service enterprises has a negative impact on cooperation among creative team members, individual idea generation and implementation, team innovation, and organizational development. In view of the importance of knowledge resource management, the prevalence of the knowledge hiding phenomenon, and the harmfulness of knowledge hiding, in recent years, academics have studied the issue of “why employees choose to hide knowledge.” It has been found that knowledge traits (Hernaus et al., 2019), individual factors such as personality traits (Anaza and Nowlin, 2017), emotional states (Aljawarneh and Atan, 2018), team and interpersonal factors (Khalid et al., 2018; Men et al., 2018; Ghani et al., 2019; He et al., 2020a), and organizational factors (Anaza and Nowlin, 2017; Jha and Varkkey, 2018) are important factors that indirectly induce and even directly cause employee knowledge hiding (He et al., 2021). Workplace stress is also one of the common challenges faced by members of organizations in contemporary society (He et al., 2020b). Therefore, scholars have begun to focus on the effects of time pressure and occupational stress on employee knowledge sharing (Marques et al., 2019), knowledge hiding (Škerlavaj et al., 2018; Feng and Wang, 2019), and silent behavior (Maqbool et al., 2019). They argue that employees have limited resources and that providing knowledge help requires additional time and energy costs. To maintain and protect existing resources, employees sometimes have to engage in knowledge hiding (Škerlavaj et al., 2018). However, the relevant studies are mainly a cursory exploration of employee knowledge hiding from the perspective of cost avoidance under time pressure. They do not provide a rigorous and complete theoretical framework for in-depth argumentation based on creative service enterprises. There is still a lack of literature on the relationship between creative time pressure and knowledge hiding from the perspective of individual resource preservation (Li et al., 2021) and team atmosphere, thereby providing an opportunity and reference for this paper to expand the antecedent influences of knowledge hiding from different theoretical foundations and from the perspective of argumentative logic.

In order to further reveal the “black box” of “work pressure → knowledge hiding,” this paper will introduce work passion and team psychological safety climate to clarify the intermediate mechanism and boundary conditions of creative time pressure acting on knowledge hiding. First, among the many antecedent variables of knowledge hiding, individual psychology is the most complex driving factor, and it is a potential research topic. However, there is a lack of research focusing on the influence of creative time pressure on knowledge hiding in creative service enterprises. As far as creative service enterprises are concerned, overtime has become the norm in creative service enterprises, and the number of tasks and limited time make employees subject to creative time pressure. Škerlavaj et al. (2018) also pointed out that time resources are among the most important resources for employees’ work in an organization. Therefore, this study explores the impact of individual psychological perception of creative time pressure on knowledge hiding at individuals’ psychological level, based on resource conservation theory.

Second, Work passion as an emotional response generated by employees in the workplace, is an emotion related to individual motivation and can be classified into harmonious and obsessive passions according to the degree to which individuals internalize external motivation (Vallerand et al., 2003). Both varieties reflect an individual’s commitment to work under the influence of external factors, and are responses and evaluations to organizational emotional events. Harmonious passion is accompanied by the spontaneous commitment of positive emotions that produce active behavioral outcomes, whereas obsessive passion is accompanied by the forced commitment of negative emotional experiences that produce passive behavioral outcomes (Vallerand et al., 2003). Harmonious and obsessive passions have also been shown to differentially influence individual knowledge-shadowing behaviors (Hua, 2021). Therefore, according to the cognitive judgment approach of affective events theory (AET), when the affective events (such as creative time pressure) are triggered, employees will first make a cognitive evaluations, that is, to evaluate the potential “gain” and “loss” that may caused by the current situation (Lazarus, 1993). Challenging or obstructive evaluation, resulting in harmonious passion (positive emotional response) or compulsive passion (negative emotional response), individuals will therefore exhibit different knowledge hiding as a result. The whole process of employees going through a complete chain system of “cognitive evaluation → emotional response → attitudes and behaviors” (Weiss and Cropanzano, 1996). However, there has been very little research on the potential competitive mediating mechanism of work passion between creative time pressure and knowledge concealment. Therefore, this study considers work passion as a mediating factor between creative time pressure and knowledge hiding.

Third, for creative service enterprises, the role of the team is clearly defined, and different team climates have an impact on employees’ behavioral performance. creative time pressure is a pressure frequently encountered by employees in creative service enterprises. Employees with different team psychological safety climate perceptions react differently when facing this pressure. Current research on the regulation mechanism of knowledge hiding focuses primarily on the individual level, and research on the team and organizational levels is lacking (Zhao and Liu, 2020). As Edmondson (2002) argued, the focus of team psychological safety climate is not on individual members within the team but on the team as a whole. Team psychological safety climate is defined as a consistent perception of the level of safety of interpersonal relationships by the team as a whole. In teams with a strong psychological safety climate, team members share a common belief that the team is safe for interpersonal risk-taking such as openly raising controversial issues and challenging each other (Tang et al., 2021), and there is a higher level of trust among team members, respect for the work product of team members, and a greater tendency to take a shared approach to problem solving. Team members help each other to bring out the strengths of the team, thereby helping to enhance team effectiveness. Therefore, this study introduces team psychological safety climate as a moderating variable to investigate the magnitude of its role in the path of creative time pressure on work passion impact knowledge-shadowing behavior, to understand more deeply the mechanism of the occurrence of this behavior.

In summary, based on the characteristics of creative service enterprises and the findings of existing studies, this study first explores the effect of creative time pressure on employees’ knowledge hiding by applying resource conservation theory. Then, using emotional event theory and a passion binary model analysis framework, the mechanism of the effect of creative time pressure on knowledge hiding is unveiled through the mediating path of work passion. Finally, combined with the team perspective, the team psychological safety climate is introduced as a boundary condition to identify the moderating effect of team psychological safety climate on the relationship between creative time pressure and knowledge hiding. This study selects as an antecedent the creative time pressure often faced by employees in creative service enterprises and extends the study of the antecedent mechanism of knowledge hiding among creative service enterprises employees by distinguishing two types of work passion and constructing a model of the dual mediating role of being regulated. It verifies the mediating role of work passion in the relationship between task characteristics and individual behaviors and extends the applicability of work passion and emotional event theories in the field of stress. It enriches the exploration of creative time pressure boundary conditions at individual and team levels and provides more precise management strategy support for suppressing knowledge-shadowing behaviors. It also provides inspiration and reference for creative service enterprises in dealing with time pressure problems and knowledge management problems.

## Theoretical background and hypotheses development

### Affective events theory

Weiss and Cropanzano (1996) proposed the AET to explore the relationship between affective events in the workplace and the affective reactions of individuals to their attitudes and behaviors. The theory suggests that the characteristics of the work environment can lead to positive or negative affective events, and the cognitive evaluation of these events can trigger the individual’s affective reactions and consequently bring about changes in the individual’s attitudes and behaviors. Affective events in this context are events that stimulate individuals to make an evaluation that produces a transient or long-lasting affective response (Weiss and Cropanzano, 1996), with positive affective events causing positive emotions and negative affective events causing negative emotions. Weiss and Cropanzano (1996) divided cognitive appraisal into primary appraisal and secondary appraisal. Primary appraisal is concerned with whether the event is consistent with one’s goals, values, or conflicts and whether the event is beneficial to one. Secondary appraisal is to assess whether the individual has sufficient resources to cope with the event (Huang et al., 2019). Affective response is the core of affective event theory (Nichola, 1993), which refers to a series of psychological, cognitive, and motivational responses of individuals to specific affective events (Mayer et al., 1990), and individuals’ affective reactions to workplace events largely determine work attitudes and behaviors (Carlson et al., 2011). The type, intensity, and duration of affective responses vary depending on the processing of a particular event, and these affective responses lead to corresponding workplace behaviors (Gray et al., 2001).

Since AET was proposed, it has been widely recognized and applied in the field of organizational behavior (Cropanzano et al., 2017; Mitchell et al., 2019) and has made important contributions to explaining emotional response triggers and outcomes at work. Specifically in this research situation, how employees go about evaluating the affective event of creative time pressure can have an impact on subsequent affective reactions and behaviors. According to affective event theory, employees evaluate the “gain” and “loss” triggered by the current creative time pressure, resulting in two different cognitive evaluations: challenging and threatening. The former focuses on the growth, gains, and positive emotional experiences that individuals may gain from stress, which is related to convergence motivation; the latter places more emphasis on the possible losses, harms, and negative emotional experiences that stress may bring, which is related to avoidance motivation. Under different evaluations, employees’ different work passion responses will be stimulated, which will then have an impact on employees’ knowledge hiding behavior. Therefore, based on the “cognitive-affective response-attitude and behavior” framework of AET, this paper investigates how employees’ evaluation of creative time pressure affects their knowledge hiding behavior by influencing their work passion after the emotional event of creative time pressure is triggered.

### Creative time pressure and knowledge hiding

Creative time pressure is one of the common challenges faced by employees in creative service enterprises. From an emotional perspective (Svenson and Edland, 1987), creative time pressure refers to a specific form of time pressure explicitly related to creativity, a stressful emotional experience in which employees feel they do not have enough time to develop creative ideas at work (Major et al., 2002; Sijbom et al., 2017), and it is a factor that makes good performance important in a given situation (Baumeister, 1984). From a resource perspective, chronic overload stress can cause employees to be continuously depleted in situations where cognitive resources are depleted and not restored, especially among those who lack resources (Schaufeli et al., 2009). Creative employees who experience creative time pressure may be reprimanded and punished for not completing creative tasks on time, leading to frustration, pain, or anger. These negative emotions will seriously deplete employees’ psychological resources. According to resource conservation theory, individuals have a strong intrinsic motivation to acquire, maintain, and protect resources and are very sensitive to resource depletion. When experiencing depletion of psychological resources, employees will try to take measures to prevent the loss of resources to avoid falling into a loss spiral (McCullough et al., 2001). Therefore, the “resource strain” caused by stressful work situations is likely to force employees to choose to hide their knowledge in the face of requests from others to mitigate the continuous loss of individual resources.

Creative time pressure, as a challenging stressor, requires employees to learn knowledge skills, complete heavy tasks, catch up on work, and take on significant responsibilities. Faced with creative time pressure as a creativity-related work requirement, creative service enterprises employees may need to continuously redouble their efforts to meet such requirements. They may need to sacrifice rest time to work overtime and to concentrate intensely at work (Li et al., 2021). Inevitably, this consumes a lot of employees’ time and energy, putting them in a resource-strained or even resource-depleted state from an overall perspective. To save time and energy for subsequent work, they are forced to choose to hide in the face of colleagues’ knowledge requests. Employees facing challenging pressures sometimes have no wish to hide their knowledge. They may choose knowledge hiding simply because they lack the time and energy to share their knowledge and are obliged to shirk or to play dumb (Škerlavaj et al., 2018). Therefore, this study argues that employees facing creative time pressure are more likely to engage in knowledge hiding. Based on the above analysis, we propose:

**Hypothesis 1:** Creative time pressure perceived by employees of creative service enterprises is positively related to knowledge hiding.

### The dual-path role of harmonious passion and obsessive passion

Affective events theory (Weiss and Cropanzano, 1996) suggests that affective events (e.g., creative time pressure) influence individuals’ behavioral performance by triggering emotional responses. For example, Widmer et al. (2012) demonstrated that time pressure reduces individuals’ positive affective experiences. However, Tadić et al. (2014) found that time pressure increases individuals’ positive affective experiences. Beck and Schmidt (2013) found that time pressure enhances individuals’ avoidance of goal orientation, but Yi et al. (2018) demonstrated that time pressure enhances individuals’ internal motivation. It is not difficult to establish that creative time pressure, as a stimulus (McGrath, 1976), will cause physiological responses, and employees, when faced with stressful situations, make challenging and threatening evaluations of the stress they face (Lazarus, 1993). As affective events that stimulate individuals to make evaluations and produce brief or long-lasting emotional responses, creative time pressure may simultaneously have positive or negative effects on affect or motivation, and different affects or motivations have different effects on individual behavior. On the one hand, creative time pressure, as a challenging stressor, motivates employees to search actively for knowledge and skills that are conducive to task completion and enhance autonomous motivation (Anwar, 2017). The resulting work passion generates excitement and work energy (Philippe et al., 2009) and employees spontaneously commits to it, who experience positive affective experiences, forming harmonious passions that lead to positive outcomes such as happiness and extra-role behaviors. On the other hand, when the individual hates the current job but has to commit to it for some reason (e.g., economic factors), they undergo negative affective experiences. When individuals hate their current job but have to engage in it for some reason (e.g., economic factors), they experience negative affective experiences and develop obsessive passions that lead to negative outcomes, such as burnout and dissatisfaction (Vallerand et al., 2010). Both passions arise from the process of internalizing external motivation, and both reflect the individual’s commitment to work, reflecting the “no pressure, no motivation” perspective in companies. However, individuals who developed the two passions experienced different emotional experiences, and there were differences in their subsequent behavioral performance, reflecting the pathways of “gain” and “loss,” respectively.

When employees perceive creative time pressure as a challenging work requirement, they identify with it and internalize it spontaneously, forming a harmonious passion; harmonious passion implies that employees have a higher quality of work passion, leading to positive outcomes such as work happiness (Vallerand, 2012), innovative exploratory behavior (Song et al., 2020), and adaptive behavior (Vallerand et al., 2010). Knowledge hiding is mostly influenced by employees’ negative emotions, which increase the likelihood of employees’ negative actions (Zhao and Xia, 2019), predicting, to some extent, that employees in creative service enterprises are more inclined to knowledge hiding when faced with others’ knowledge seeking. In contrast, harmonious passion brings employees a more positive emotional experience, emotionally triggering knowledge-sharing behavior (Anwar, 2017) and reducing the knowledge hiding of core employees. Hence, we propose the following hypothesis:

**Hypothesis 2a:** Creative time pressure indirectly reduces employee knowledge hiding through the mediating effect of harmonious passion.

However, when individuals perceive creative time pressure as an excessive workload and do not want to recognize it, but they are afraid of punishment for not completing the task, they force themselves to act, and they develop compulsive passion. Individuals who develop compulsive passion also develop a commitment to their work. However, this commitment is not spontaneously formed, does not sufficiently indicate self-identification with the work, and frequently indicates a compulsion to act for self-protective purposes (Curran et al., 2015). According to the stressor-stress theory, stressors cause stressful processes in individuals, causing them to feel more anxiety, tension, exhaustion, and other forms of stress, eventually leading to a series of outcomes, such as negative affect (Vallerand et al., 2003), withdrawal behavior at work, job burnout, and counterproductive behavior. Therefore, obsessive passions can produce more negative emotions than harmonious passions. Individuals experiencing obsessive passions will devote their energy to meeting external demands quickly. They will not be able to work with the same degree of autonomy as those experiencing harmonious passions. They often create work conflicts between multiple tasks (Vallerand, 2012) and do not have time to consider the knowledge requests of others during interactions with them. Therefore, they choose knowledge hiding. Obsessive passions, while keeping employees engaged in a task, can also make individuals feel more stressed because people tend to deal with stressful situations by acquiring, protecting, and retaining resources (Hobfoll, 1989). Therefore, obsessive, passionate employees are more inclined to consider knowledge as an important personal resource for knowledge hiding. Therefore, we propose the following:

**Hypothesis 2b:** Creative time pressure indirectly increases employee knowledge hiding through the mediating effect of obsessive passion.

### The moderating effect of team psychological safety climate

The concept of psychological safety emerged from the study of organizational change (Schein and Bennis, 1967). At the individual level, psychological safety is the individual’s cognitive evaluation and experience of the work environment (Edmondson, 1999). As research has progressed, the concept of psychological safety has expanded. At the team level, psychological safety is a shared belief among team members that it is safe to take risks (Edmondson, 1999). In teams with a high psychological safety climate, employees can feel organizational support, mutual trust, and respect among members, and members’ anxiety about possible embarrassing or threatening reactions from colleagues is reduced (Baer and Frese, 2002). Employees are more actively engaged in their work and learning (Spreitzer et al., 2012). A study by Shang et al. (2019) found that employees who are passionate about innovation have a stronger willingness to share knowledge and are more likely to share knowledge skills and new ideas when they feel a higher level of security and trust.

Based on the findings of affective event theory and self-determination theory, we concluded that employees with different perceptions of team psychological safety climate would have different emotional reactions when faced with creative time pressure. First, according to emotional event theory, an individual’s perception of a work event triggers corresponding emotions, changing the individual’s behavior. Employees with high team psychological safety climate perceptions are more open to challenges (Liberman et al., 2001). Engagement with tasks and intrinsic motivation for self-improvement are stronger, allowing them to fully engage in their work (Grund and Fries, 2018); the challenges of time pressure are more aligned with their intrinsic motivation and are thus more easily internalized by these employees. Second, a team psychological safety climate promotes organizational citizenship behaviors, and good and calm emotions enable employees to have a positive perception of their environment and to be more likely to be proactive in helping the organization and others. Finally, according to self-determination theory, internal motivation and external motivation are more likely to be internalized when the three major psychological needs of individuals—competence, autonomy, and relationships—are met. Team psychological safety climate facilitates this process to an extent. It increases employees’ perceptions of autonomy (Kahn, 1990) while reducing their concerns about interpersonal conflict (Zhang et al., 2015), making employees willing and able to focus more attention on constructive thinking and problem-solution-seeking. Team psychological safety climate promotes information sharing, encourages risk-taking behavior, and prevents individuals from fearing complaints, rejection, punishment, or exploitation by the opportunistic behavior of others as a consequence of knowledge sharing (Edmondson, 1999), encouraging employees to focus on positive social exchange. In contrast, employees with low team psychological safety climate perceptions see things differently. They are reluctant to accept complex goals, are more sensitive to negative information in the environment (e.g., obligations, punishments) (Lanaj et al., 2012), and take actions aimed primarily at avoiding negative outcomes. Based on the above analysis, this study concludes that employees with high team psychological safety climate perception are more likely to perceive the positive components of time pressure and identify more with the challenges posed by time pressure, thereby developing harmonious passion. Thus, team psychological safety climate significantly moderates the relationship between creative time pressure and harmonious passion.

**Hypothesis 3a:** When employees perceive a high team psychological safety climate, the positive relationship between creative time pressure and harmonious passion becomes stronger; when employees perceive a low team psychological safety climate, the positive relationship between time pressure and harmonious passion becomes weaker.

Conversely, employees with low team psychological safety climate perception are more likely to perceive the negative components of time pressure; they are reluctant to accept complex goals; they are afraid of being punished for not completing tasks; and they reluctantly accept time pressure, thereby generating obsessive passion. Therefore, team psychological safety climate significantly moderates the relationship between creative time pressure and obsessive passion.

**Hypothesis 3b:** When employees perceive high team psychological safety climate, the positive relationship between creative time pressure and obsessive passion becomes weaker; when employees perceive low team psychological safety climate, the positive relationship between creative time pressure and obsessive passion increases.

Based on the hypotheses inferred from the above analysis, this study further argues that the mediating role of harmonious and obsessive passions is also moderated by the team psychological safety climate. Specifically, when employees perceive a strong team psychological safety climate, they are more likely to identify with creative time pressure and proactively cope with it, resulting in more harmonious passion and less obsessive passion (Song et al., 2020). More harmonious passion represents more positive emotional experiences and higher perceptions of team support, reducing, to a certain extent, employees’ knowledge. Accordingly, we hypothesize that:

**Hypothesis 4a:** Team psychological safety climate positively moderates the indirect effect of creative time pressure on employees’ knowledge hiding via harmonious passion, i.e., the above indirect effect is stronger in a high team psychological safety climate.

Conversely, when employees perceive a low team psychological safety climate, they perceive creative time pressure as an external constraint and are influenced by factors such as responsibility and obligation. They feel obliged to respond to creative time pressure, resulting in more obsessive passion and less harmonious passion. More obsessive passions mean more negative emotional experiences, increased likelihood of conflicts when multitasking, and more resource retention. To some extent, this promotes the generation of core employees’ knowledge hiding (Hua, 2021). Therefore, the positive effect of creative time pressure through increasing obsessive passions and thus increasing core employees’ knowledge hiding will be diminished. Therefore, we propose the following:

**Hypothesis 4b:** Team psychological safety climate negatively moderates the indirect effect of creative time pressure on employees’ knowledge hiding via obsessive passion, i.e., the above indirect effect is diminished in a high team psychological safety climate.

**FIGURE 1:**
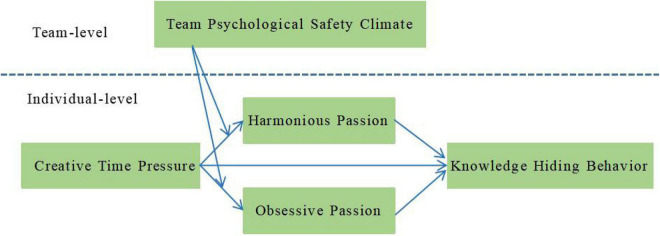
Theoretical framework.

In summary, drawing from Resource Conservation Theory and Affective Events Theory, this research examines the relationship between creative time pressure and knowledge hiding, focusing on the mediating role of work passion and the moderating effect of team psychological safety climate (see Figure 1).

## Materials and methods

### Chinese creative service enterprises context

Our empirical setting is advertising companies in creative service enterprises in China. Several criteria guided this choice. First, in the context of economic development of the new normal, China’s economic growth has slowed down, the economic growth model has shifted from speed and sloppy to quality and intensive, the industrial value chain has advanced from the manufacturing segment to R&D, design, creativity and standard segments, and the development of creative industries has become an important strategic choice for the economy to achieve new growth (Throsby, 2017). In recent years, China has invested more in creative industries, so many creative enterprises of different types and scales have emerged, and according to a survey conducted by the National Bureau of Statistics of China (NBS, 2022), in 2021, 65,000 cultural and related industry enterprises above the scale in China achieved a business income of 119,064 billion yuan, an increase of 16% over the previous year, with an average growth of 8.9% in 2 years, accounting for 10.41% of GDP, of which the cultural services industry was 5,625.5 billion yuan, an increase of 16.3% (NBS, 2022), and cultural and creative industries became a typical industry driving economic development in China (Wen, 2017), and the report “Creative Economy Outlook: Trends in International Trade in Creative Industries” published by the United Nations Trade and Development Organization (UNCTAD, 2018) shows that global trade in creative products is growing rapidly, with China dominating the trade in creative products and services (UNCTAD, 2018). This makes China a particularly suitable context.

Secondly, as one of the ten key industries of creative industries, the advertising industry has developed with strong momentum and becoming one of the core forces shaping today’s economic and cultural life. In terms of 2-year average growth rate, in 2021, the 2-year average growth rate of creative design service industry is 13.8%, which is higher than the average level of cultural enterprises. In terms of 16 sub-sectors, the 2-year average growth rate of business income of Internet advertising service industry ranks second only to the manufacturing industry of wearing intelligent cultural equipment, at 31.8% (NBS, 2022). Meanwhile, advertising is an intellectual and knowledge-based industry with talent as the main factor, and creativity is its core (West et al., 2019) and it is also the core competitiveness of advertisers. Advertisers tend to pay close attention to their creative products, whose completion often requires multiple skills, pay close attention to the uniqueness as well as the difference of their creative products, and at the same time face more severe creative time pressure than other industries, and become more cautious about knowledge sharing. Thus advertising companies provides a suitable context for investigating the effect of creative time pressure on knowledge hiding behavior.

### Sample and procedure

This study was conducted using electronic questionnaires, and the respondents came from creative employees of advertising enterprises in Guangdong, Shandong, Zhejiang, Fujian and other regions. They were concentrated in the Creative Department, Strategy/Planning Department and Media Department. The corresponding positions included copywriting, creative, design, final production, planning, media, visual effects, graphic design, etc. This study was conducted in mid-March 2022, and 417 questionnaires were collected, of which 381 were valid, an efficiency rate of 91.3%. The descriptive analysis of the sample is as follows: in terms of gender, male employees account for 42.3%, and female employees account for 57.7%; in terms of age group, employees aged 26–35 are the most numerous, accounting for 63.8% of the total, followed by those aged 18–25, accounting for 16%, and the total number of employees aged 18–35 accounts for 79.8%, showing that employees in the advertising industry are mainly young and middle-aged people; in terms of education distribution, bachelor’s degrees account for 70.6%, and master’s degree and above account for 11%, in line with the current situation in which most advertising employees are people with high levels of education; the distribution of years of service is more balanced, whereby 32.3% of the total accounts for 5–10 years, followed by 1–3 years, accounting for 22.3%; the annual income of 100–200 thousand yuan accounts for 40.4%, followed by 50 thousand yuan. The income level of employees in the advertising companies in the sample is more evenly distributed, and the distribution characteristics of high, middle, and low income levels match those of education and working years, in line with the industry characteristics whereby the advertising industry attaches importance to knowledge-creating talents; among the unit sizes, 74.5% of the respondents were from small-scale advertising enterprises with 100 employees or fewer.

### Measures

The scales used in this study were sourced from international authoritative journals that have been proven to be credible. The original scales were translated, and the wording and word order in the questions were adjusted to ensure that they conformed to local language conventions while retaining the original meaning of the items. Then, the Chinese version of the scale was back-translated to ensure that the adjusted Chinese scale was not distorted. All scales were scored on a 7-point Likert scale, from “not at all” to “completely” on a scale of 1–7. The complete measurements scales is in the Appendix.

#### Creative time pressure

Creative time pressure was measured using Baer and Oldham’s (2006) scale, which contains five questions such as “I don’t have time to think of new ideas.”

#### Work passion

Work passion was measured using the scale developed by Vallerand et al. (2003), which contains 14 items, the first seven of which measure harmonious passion, such as “My work brings me a variety of experiences.” The second seven items measure obsessive passion, such as “I can’t live without work.”

#### Knowledge hiding

Knowledge hiding uses a scale developed by Connelly et al. (2011), which contains three dimensions, “promised to help but did not really intend to do so” (evasive hiding), “will pretend you don’t know the information” (playing dumb), and “will explain that your duties do not allow you to tell them” (rationalized hiding), a total of 12 questions.

#### Team psychological safety climate

Team psychological safety climate is a scale developed by Edmondson (1999), which includes five questions such as “Even if I make a mistake at work, I will not complain about it.”

#### Control variables

Several variables were controlled. Employees’ gender, age, education, years of service, income, and company size often affect certain organizational behavior outcome variables (Robbins and Judge, 2019). In this paper, they are treated as control variables.

## Results

### Reliability and validity tests

Reliability analysis of the variables was conducted using SPSS 21.0. As Table 1 shows, the Cronbach’s alpha coefficient and composite reliability (CR) for all variables were greater than 0.7, indicating a high level of reliability of the questionnaire and a high internal consistency and stability of the scale. Therefore, the variables measured in this study can be considered to have good reliability. In addition, the factor loadings of the corresponding measures of each variable were all greater than the criterion of 0.5, and the AVE (average variance extracted) values of each variable exceeded the criterion of 0.5. This indicates that all the variables involved in this study have good convergent validity.

**TABLE 1:** Indices for construct reliability and convergent validity.

Construct	Item	Factor loading	Cronbach’s alpha	CR	AVE
Creative time pressure (CTP)	CTP1	0.831	0.893	0.894	0.628
	CTP2	0.786			
	CTP3	0.781			
	CTP4	0.737			
	CTP5	0.823			
Harmonious passion (HP)	HP1	0.833	0.937	0.937	0.680
	HP2	0.858			
	HP3	0.805			
	HP4	0.8			
	HP5	0.809			
	HP6	0.834			
	HP7	0.831			
Obsessive passion (OP)	OP1	0.852	0.907	0.907	0.585
	OP2	0.694			
	OP3	0.702			
	OP4	0.678			
	OP5	0.792			
	OP6	0.775			
	OP7	0.841			
Knowledge hiding (KH)	KH1	0.8	0.948	0.948	0.605
	KH2	0.783			
	KH3	0.779			
	KH4	0.775			
	KH5	0.796			
	KH6	0.768			
	KH7	0.761			
	KH8	0.768			
Knowledge hiding (KH)	KH9	0.782	0.948	0.948	0.605
	KH10	0.774			
	KH11	0.743			
	KH12	0.805			
Team psychological safety climate (TPSC)	TPSC1	0.854	0.919	0.919	0.695
	TPSC2	0.817			
	TPSC3	0.808			
	TPSC4	0.816			
	TPSC5	0.872			

This paper used AMOS 21. 0 to conduct validated factor analysis on the study variables to determine that the variables were not identical constructs Table 2 shows the results of the validated factor analysis (CFA). The results show that the five-factor model (CTP, HP, OP, KH, TPSC) had the best fit with the observed data (χ2/df = 1.622, TLI = 0.959, CFI = 0.962, and RMSEA = 0.04), indicating that the five-factor hypothesis model of this study has high discriminant and structural validity.

**TABLE 2:** Results for confirmatory factor analysis.

Model	*X*^2^/df	GFI	AGFI	NFI	IFI	TLI	CFI	RMSEA
Five-factor model	1.622	0.884	0.868	0.908	0.963	0.959	0.962	0.04
Four-factor model	3.558	0.707	0.668	0.797	0.845	0.833	0.845	0.082
Three-factor model	6.348	0.514	0.453	0.636	0.675	0.652	0.673	0.119
Two-factor model	8.921	0.384	0.308	0.487	0.516	0.484	0.514	0.144
Single-factor model	10.915	0.308	0.224	0.371	0.393	0.354	0.391	0.162

Single-factor model: CTP + HP + OP + KH + TPSC; Two-factor model: CTP + HP + OP + KH, TPSC; Three-factor model: CTP + HP + OP, KH, TPSC; Four-factor model: CTP + HP, OP, KH, TPSC; Five-factor model: CTP, HP, OP, KH, TPSC.

### Common method deviation analysis

The widely used Harman’s single-factor method was used to test for common method bias before data analysis to control for common method bias (Podsakoff et al., 2003). All scale items were analyzed together in exploratory factor analysis. According to the test data, five common factors with eigenvalues greater than 1 were extracted, and the cumulative variance explained was 68.898%. The first factor explained 28.971%, less than 40%. Therefore, there was no one common factor explaining most of the variance. According to the results in Table 2, the single-factor model had a poor fit; in comparison, the five-factor model fit indicators were better. This indicates that there is no significant common method bias in the study.

### Correlation analysis

Correlation analysis of each variable was conducted using SPSS 21.0. As Table 3 showed that the correlation coefficients of creative time pressure, harmonious passion, compulsive passion, team psychological safety climate, and knowledge hiding were 0.350, −0.311, 0.244, and 0.247, respectively. The corresponding *p*-values were less than 0.01, making them statistically significant, indicating that creative time pressure, harmonious passion, obsessive passion, team psychological safety climate, and knowledge hiding were all significantly correlated, laying a good foundation for the next mediating effect test.

**TABLE 3:** Coefficients of variables.

Variables	CTP	HP	OP	TPSC	KH
Creative time pressure (CTP)	1				
Harmonious passion (HP)	0.194**	1			
Obsessive passion (OP)	0.419**	0.099	1		
Team psychological safety climate (TPSC)	0.509**	0.233**	0.532**	1	
Knowledge hiding (KH)	0.350**	−0.311**	0.244**	0.247**	1

**p < 0.01.

### Direct and mediated effects tests

Regression analysis was conducted using SPSS 21.0 to test the hypotheses (see Table 4). After controlling for gender, age, education, annual income, working years, and company size, creative time pressure had a significant positive effect on knowledge hiding (Model 1: β = 0.342, *p* < 0.001), thereby supporting Hypothesis 1. In model 2, after adding an independent variable (creative time pressure) and mediating variables (harmonious passion and obsessive passion), the regression coefficient of the independent variable (creative time pressure) on the dependent variable (knowledge hiding) was β = 0.370, *p* < 0.001. The regression coefficient of the mediating variable (harmonious passion) on the dependent variable (knowledge hiding) was β = −0.410, *p* < 0.001. The regression coefficient of (obsessive passion) on the dependent variable (knowledge hiding) was β = 0.118, *p* < 0.05. Therefore, it can be determined that harmonious passion and obsessive passion have a partial mediating effect on creative time pressure and knowledge hiding. Therefore, we have initial evidence supporting Hypotheses 2a and 2b.

**TABLE 4:** Results of hierarchical regression analysis of mediation and moderation hypotheses.

Variables		Knowledge hiding (KH)		Harmonious passion (HP)		Obsessive passion (OP)	
		Model1	Model2	Model3	Model4	Model5	Model6
Control variables	Gender	0.053	0.021	−0.057	−0.063	0.082	0.083
	Age	−0.026	0.010	0.104	0.075	0.051	0.030
	Education	0.091	0.101	0.036	0.017	0.030	0.031
	Working years	0.014	−0.016	−0.110	−0.069	−0.118	−0.100
	Company sizes	−0.017	0.000	0.041	0.035	−0.010	−0.004
	Annual income	−0.023	−0.062	−0.080	−0.070	0.051	0.058
Independent variables	CTP	0.342***	0.370***	0.188***	0.170**	0.404***	0.146**
Mediator	HP		−0.401***				
	OP		0.118*				
Moderator	TPSC				0.194**		0.410***
Interaction effects	CTP × TPSC				0.193***		−0.128**
*R* ^2^		0.135	0.135	0.056	0.108	0.191	0.338
Δ*R*^2^		0.296	0.161	0.056	0.052	0.191	0.147
*F*		8.308***	17.341***	3.171***	4.974***	12.611***	21.043***

CTP, creative time pressure; TPSC, team psychological safety climate.

*p < 0.05, **p < 0.01, ***p < 0.001.

### Moderating effect test

In Table 4, the model CTP × TPSC interaction term has a significant positive effect on harmonious passion (β = 0.193, *p* < 0.001); and model 6, CTP × TPSC has a significant negative effect on obsessive passion (β = −0.128, *p* < 0.001). Therefore, Hypotheses 3a and 3d are fully supported.

**FIGURE 2:**
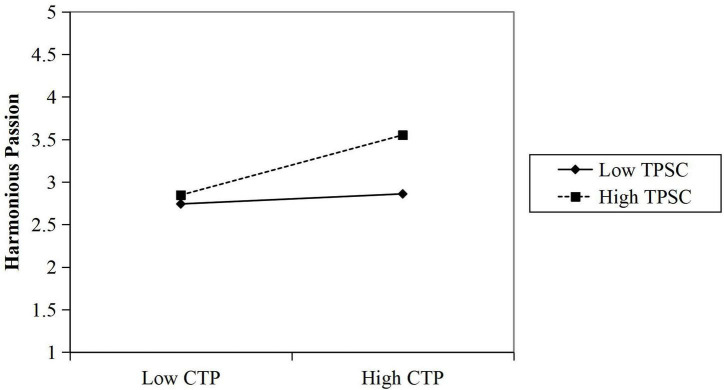
The moderating effect of team psychological safety climate (TPSC) on the relationship between creative time pressure (CTP) and harmonious passion.

**FIGURE 3:**
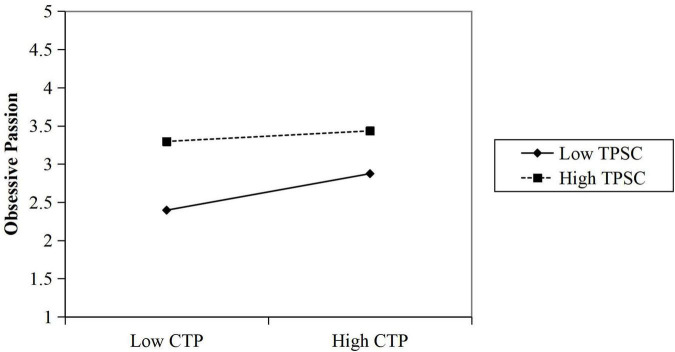
The moderating effect of team psychological safety climate (TPSC) on the relationship between creative time pressure (CTP) and obsessive passion.

The significance of the interaction between creative time pressure and harmonious and obsessive passions was tested using the simple slope method (the benchmark is the mean of ± 1 standard deviation) to explore the differences in the effects of creative time pressure on harmonious and obsessive passions at different levels of team psychological safety climate. The moderating effect was plotted in Figures 2, 3 to show the moderating effect more visually. The slope of the two lines in Figure 2 shows that the positive effect of creative time pressure P on harmonious passion is greater when the team psychological safety climate is high than when the team psychological safety climate is low. The comparison of the slope of the two lines in Figure 3 shows that the negative effect of creative time pressure on obsessive passion is greater when the team psychological safety climate is low than when the team psychological safety climate is high.

### Moderated mediating effects test

When the process method was used to examine the mediated effects of being moderated (see Table 5), there was a positive significant indirect effect of creative time pressure on knowledge hiding mediated by harmonious passion in the condition of differences in high and low levels of team psychological safety climate. There was a significant indirect effect difference (Δγ = −0.068, *p* < 0.01). Under the condition of high and low levels of team psychological safety climate, there was a positive and significant indirect effect of creative time pressure on knowledge concealment mediated by obsessive passion, and there was a significant indirect effect difference (Δγ = −0.012, *p* < 0.01). Therefore Hypotheses 4a and 4b were supported.

**TABLE 5:** Moderated mediation test results.

IV	Moderator	Conditional indirect effects	LL 95%CI	UL 95% CI
**CTP→HP→KH**				
CTP	Low TPSC(−SD)	−0.005	−0.055	0.045
	High TPSC(+SD)	−0.124***	−0.205	−0.055
	Difference	−0.068***	−0.115	−0.027
**CTP→OP→KH**				
CTP	Low TPSC(−SD)	0.026***	0.002	0.067
	High TPSC(+SD)	0.004	−0.011	0.031
	Difference	−0.012***	−0.04	−0.001

CTP, creative time pressure; OP, obsessive passion; HP, harmonious passion.

## Discussion

Based on resource conservation theory and emotional event theory, this study distinguishes work passions into harmonious passions and obsessive passions. On this basis, 381 Chinese advertising agency employees were used as a sample to construct and explore the effects of creative time pressure via the two types of work passion on knowledge hiding and their underlying mechanisms of action. The empirical method was used to reach the following conclusions: (1) creative time pressure positively affects employees’ knowledge hiding. That is, the greater the creative time pressure, the more likely employees are to engage in knowledge hiding. (2) Both harmonious passion and obsessive passion play a partial mediating role in the relationship between creative time pressure and knowledge hiding. (3) Team psychological safety climate moderates the mediating role of work passion in the relationship between creative time pressure and knowledge hiding. Under the perception of high team psychological safety climate, the indirect effect of creative time pressure on the relationship between employees’ knowledge hiding via harmonious passion was enhanced, and the indirect effect of creative time pressure on the relationship between employees’ knowledge hiding via obsessive passion was weakened.

First, we found that the creative time pressure employees perceive in their workplace increases their knowledge hiding. Individuals who perceive high creative time pressure will be more focused on their tasks. As a result, they allocate fewer cognitive resources to consider the reasons for their colleagues’ requests for assistance. In other words, individuals facing knowledge demands are more inclined to prioritize their own tasks and are less concerned with their peers’ tasks. Even if the request is commendable and long-term compliance with the request is beneficial to the organization and the employee, these benefits will not be given consideration, and employees under time pressure will hide knowledge. As creative time pressure increases, employees reduce the loss of other resources by hiding their knowledge.

Second, this study verified the mediating role of harmonious and obsessive passions based on affective event theory and the passion binary model. The empirical results show that harmonious passion and obsessive passion are the “bridges” between creative time pressure and knowledge hiding. When employees experience creative time pressure, they first produce emotional reactions and then change their behaviors. Passion for work is a motivation-related emotional experience that is influenced by external factors (e.g., creative time pressure). Whether harmonious passion or obsessive passion is formed, an individual’s commitment to work is increased, reflecting the connotation of “no pressure, no motivation.” It was found that the mediating role of harmonious passion in the relationship between creative time pressure and knowledge hiding and the mediating role of obsessive passion in the relationship between creative time pressure and knowledge hiding were consistent with the hypothesis that harmonious passion could reduce employees’ knowledge hiding, while obsessive passion could enhance knowledge hiding. The reason for this may be that under creative time pressure, employees’ identification with their work will stimulate their spontaneous work enthusiasm, and they believe they can demonstrate their ability at work and are, therefore, more willing to share their knowledge. However, when employees perceive excessive creative time pressure, they are unwilling to identify with it, and they generate time anxiety. They feel obliged to complete the work, causing them to experience negative emotional experiences and to generate obsessive passion. Core employees experiencing obsessive passion are more inclined to consider knowledge as an important personal resource and are therefore more reluctant to share their knowledge with others.

Finally, this study also examined the moderating effect of team psychological safety climate. The results show that when employees perceived a strong team psychological safety climate, creative time pressure was more likely to trigger harmonious passions and, in so doing, reduce knowledge hiding. When employees perceived a low team psychological safety climate, creative time pressure was more likely to trigger their obsessive passions and further increase knowledge hiding. This finding also explains, to some extent, the common phenomenon that individuals facing the same or almost the same time pressure in different teams in the same company perform very differently. The team psychological safety climate affects their perception of creative time pressure, causing their emotional responses to time pressure to differ, changing their subsequent attitudes and behaviors.

### Theoretical implications

First, this study broadens the research related to creative time pressure and knowledge hiding. The discussion of whether creative time pressure, as one of the pressures frequently faced by employees in creative service enterprises, can stimulate employees’ passion for work and maximize their knowledge value could enrich the study of the positive effects of creative time pressure. This paper also takes creative time pressure as an antecedent and confirms that it positively affects knowledge hiding, clearly explaining the inner mechanism of knowledge hiding generation among creative service enterprises employees, responding to the call of scholars in the field of knowledge management, and enriching the theoretical research related to knowledge hiding.

Second, this study introduces the competing mediating mechanisms of harmonious and obsessive passions to explore in depth the mechanism of action of creative time pressure on knowledge hiding. Few previous studies have explored the mechanism of action in the relationship between creative time pressure and knowledge hiding from a dual-path perspective. To this end, this study focuses on two different emotional experiences of individuals at work: harmonious passion and obsessive passion. It provides an in-depth analysis of the mechanism of the effect of time pressure on knowledge hiding, further explaining the reasons for the debate on the relationship between time pressure and knowledge hiding. In addition, the mediating role of work passion in the relationship between task characteristics and individual behavior has been confirmed by several scholars (Liu et al., 2011). However, this mechanism has not been applied to research in the field of stress. The present study confirmed the mediating role of work passion in the relationship between time pressure and knowledge hiding, extending the applicability of work passion and affective event theory within the stress domain.

Finally, by examining the moderating role of team psychological safety climate in conjunction with the team perspective, boundary conditions were identified for changing the role of time pressure in the effect of knowledge hiding. The influence of internal team factors on employees is highlighted. Conversely, the viewpoint of emotional event theory states that work events will change individuals’ behaviors by triggering emotions, which is consistent with the findings of this study that when employees perceive a high team psychological safety climate, creative time pressure is more likely to trigger the action path of harmonious passion, and knowledge hiding is inhibited as a result. However, when employees perceive a low team psychological safety climate, creative time pressure is more likely to trigger the path of obsessive passion, thereby enhancing knowledge hiding. The findings of this study not only verify the views of affective event theory but also identify the team and organizational characteristics that change the effect of creative time pressure, further exploring the mechanism of the effect of creative time pressure on employees’ knowledge hiding and answering the question as to how creative time pressure affects employees’ knowledge hiding and under what conditions.

### Practical implications

Our findings offer several managerial implications for avoiding knowledge hiding between coworkers in creative service enterprises. First, managers should pay attention to time pressure and should allocate tasks scientifically and effectively according to the priority of the tasks. For tasks with low time requirements, managers should give more autonomy to the employees. They should exercise employees’ abilities, help them achieve self-improvement, and stimulate their vitality and enthusiasm. For urgent tasks, managers should give employees appropriate consideration, while providing guidance and assistance, giving emotional reassurance, and guiding them to improve efficiency through knowledge sharing. Managers should also be alert to the negative effects of time pressure so that they can avoid the appropriation of employees’ physical and mental resources due to excessive pursuit of efficiency, resulting in hidden knowledge. Therefore, managers should establish a people-oriented management consciousness and provide time and space for employees to communicate and share. For example, building an enterprise knowledge base, reducing the time required to respond to employees’ knowledge requests, providing break areas and unstructured social time (employees take breaks at the same time instead of individually), and increasing the benign exchange of knowledge to improve the operational efficiency of the enterprise.

Second, managers should attend to the emotional response of employees and focus on the stimulation and protection of employees’ harmonious passion. The work passion triggered by creative time pressure is the autonomous internalization or passive internalization of their motivation, and harmonious passion can bring more desirable results. Managers need to think about the problem of how to enhance the promotion by creative time pressure of harmonious passion and reduce the promotion of obsessive passion causes. Managers can help employees turn time pressure into motivation by enhancing their harmonious passion through behaviors such as emotional support, empowerment, and assistance.

Finally, managers need to attend to the creation of a safe atmosphere in the team. Under time pressure, employees in a strong team safety atmosphere are better able to cope with creative time pressure. Companies should respect employees’ opinions, value fairness, and build a sharing and inclusive corporate culture. Team leaders can also establish a dedicated platform for sharing issues and exchanging information, through which employees can express and publish their ideas and opinions and seek communication and explanation from relevant team leaders. The above communication methods can effectively counteract employees’ worries and concerns, enhance employees’ psychological security perceptions, and achieve effective motivation for employees.

### Limitations and future research

Although the hypotheses of this study are confirmed, there are still improvements that could be made, mainly in the following aspects: First, this study uses a single point in time to collect data. A multi-source and multi-stage tracking method could be used to collect data and predict individual behavior more effectively. Second, the sample in this study was composed mainly of employees of advertising agencies, and although the employees of advertising agencies frequently engage in creative activities in their work, creative activities are not limited to the advertising industry. The findings of this study based on the above-mentioned industry may be affected when generalizing to other industries. In future studies, the sample should be expanded to include more industries to enhance the generalizability of the findings. Again, our focus on the Chinese context could also limit the generalizability to other countries. employees’ perceptions and evaluations of time pressure is culture-specific. For example, in organizations that emphasize collectivism, and ethics, employees with high pro-organizational motivation may feel that time pressure is a positive stressor, so it has less impact on knowledge hiding behavior. In view of this, in-depth comparative study of time pressure and knowledge hiding behaviors undercreative service enterprises between Chinese and employees of other cultural backgrounds calls for more attention. Finally, this study only examined the moderating effect of team characteristics on the role of time pressure. Studies in the field of stress also point out that leaders are important situational factors that alter employees’ perceptions of stress (Maruping et al., 2015) and that situational factors and individual characteristics jointly influence employees’ responses to stress (Song et al., 2020). Therefore, future research could attempt to construct a more comprehensive model of the mechanism of action, such as a dual mediating role that includes a triple interaction, to gain a deeper and more comprehensive understanding of the role of time pressure on innovation behavior.

## Data availability statement

The original contributions presented in this study are included in the article/supplementary material, further inquiries can be directed to the corresponding author/s.

## Author contributions

XC, WL, and AX designed and supervised the study and wrote the manuscript. XC collected the data. XC and WL analyzed the data. All authors contributed equally to this manuscript, reviewed, and approved this manuscript for publication.

## Conflict of interest

The authors declare that the research was conducted in the absence of any commercial or financial relationships that could be construed as a potential conflict of interest.

## Publisher’s note

All claims expressed in this article are solely those of the authors and do not necessarily represent those of their affiliated organizations, or those of the publisher, the editors and the reviewers. Any product that may be evaluated in this article, or claim that may be made by its manufacturer, is not guaranteed or endorsed by the publisher.

## Appendix

### Appendix 1: Measurement Scale

**Creative time pressure**

(1) I don’t have time to think of new ideas.

(2) I don’t have much time for thinking up wild ideas ——I am too busy just getting my job done.

(3) Coming up with new ideas always takes too much time.

(4) Executing new ideas always takes a lot of time.

(5) I don’t have time to implement new ideas.

**Work PassionHarmonious Passion**

(1) My work brings me a variety of experiences.

(2) I appreciate new things discovered at work.

(3) The work gave me an unforgettable experience.

(4) My personal strengths are reflected at work.

(5) My work is in harmony with other activities in my life.

(6) Even though work is a passion for me, I’m still in control.

(7) I love this job very much.

**Obsessive Passion**

(8) I can’t live without work.

(9) The desire to work is so strong, I can’t help myself.

(10) It’s hard to imagine my life without a job.

(11) I am emotionally dependent on work.

(12) I have difficulties controlling my urge to do my work.

(13) I have a feeling of being controlled by my work.

(14) Whether I can go to work determines my emotional state.

**Knowledge Hiding**

When colleagues ask you for work methods, techniques or asks you to share your work reports, templates or tools and other knowledge with them, you may

(1) Promise to help but do not really intend to do so.

(2) Agree to help but give different information than they demand.

(3) Tell that you will help later, but delay as much as possible.

(4) Give something other than what they really demand.

(5) Pretend you don’t know the information.

(6) Even though you know, you will say you don’t know.

(7) Pretend not to know what they are talking about.

(8) Say that you do not know the information very well.

(9) Explain that your duties do not allow you to tell them.

(10) Explain that the information is confidential and only available to specific interested parties.

(11) Say that the leader does not allow everyone to pass this message.

(12) Directly say you can’t tell them.

**Team Psychological Safety Climate**

(1) Even if I make a mistake at work, I will not complain about it.

(2) I can raise problems and difficulties in my work.

(3) In our company, no one will deliberately discredit my efforts.

(4) It is easy for me to seek help from other colleagues in the company.

(5) My unique skills and talents are reused in the process of collaborating with colleagues.
